# Preoperative Use of Intravenous Contrast Media Is Associated With Decreased Excellent Response Rates in Intermediate-Risk DTC Patients Who Subsequently Receive Total Thyroidectomy and Low-Dose RAI Therapy

**DOI:** 10.3389/fonc.2020.01297

**Published:** 2020-09-15

**Authors:** Wei Lan, Wang Renjie, Wan Qichang, Teng Feiyue, Ma Qingjie, Ji Bin

**Affiliations:** ^1^Department of Nuclear Medicine, China-Japan Union Hospital of Jilin University, Changchun, China; ^2^NHC Key Laboratory of Radiobiology, School of Public Health of Jilin University, Changchun, China; ^3^Department of Nuclear Medicine, Third Affiliated Hospital of Sun Yat-sen University, Guangzhou, China

**Keywords:** differentiated thyroid cancer, intravenous contrast media, radioactive iodine therapy, delay time, excellent response

## Abstract

**Purpose:** To evaluate the impact of preoperative use of intravenous contrast media (ICM) on the excellent response (ER) rates in a cohort of intermediate-risk differentiated thyroid cancer (DTC) patients who received total thyroidectomy (TT) and low-dose radioactive iodine (RAI) therapy.

**Methods:** A total of 683 consecutive patients were retrospectively reviewed in a single center between August 2016 and August 2018. Patients were divided into ICM group (*n* = 532) and non-ICM group (*n* = 151). Intravenous contrast media patients were 1:1 propensity matched to non-ICM patients based on T stage, N stage, and urinary iodine. Risk-adjusted logistic regression models were constructed to assess the association between the use of ICM and ER rates.

**Results:** Intravenous contrast media patients had significantly higher T stage (*P* < 0.001), N stage (*P* < 0.001), urinary iodine (*P* < 0.001), and ps-Tg (*P* = 0.042) than non-ICM patients. Preoperative use of ICM was found to be significantly associated with decreased ER rates in both the primary cohort [odds ratio (OR) = 0.47, 95% confidence interval (CI) = 0.32–0.71; *P* < 0.001] and the matched cohort (OR = 0.48, 95% CI = 0.25–0.94; *P* = 0.031). Subgroup analysis on RAI delay time in the primary cohort revealed that ER rates in ICM patients were significantly lower than that of non-ICM patients for 1–2 months (*P* = 0.0245) and >2–3 months (*P* = 0.0221) subgroups, but not for >3–4 months, >4–5 months, and >5–6 months subgroups (all *P* > 0.05). A delay time of >3–4 months exhibited the highest ER rate (63.08%) within the ICM group.

**Conclusions:** Preoperative use of ICM is associated with decreased ER rates in intermediate-risk DTC patients who subsequently receive TT and low-dose RAI therapy. For such patients, if ICM has already been received, an RAI delay time of >3–4 months would seem to be more appropriate to achieve better ER rates.

## Introduction

Post-operative use of radioactive iodine (RAI) continues to be conservative in differentiated thyroid cancer (DTC) patients with low to intermediate recurrence risk. While high dose is considered to be associated with dysfunctions in non-thyroidal organs such as salivary and lachrymal and long-term effects such as second primary cancer, plenty of studies have demonstrated that low dose is as effective as high dose in achieving ablation success and controlling disease recurrence in this patient population (1–3). However, delivering sufficient absorbed doses to the thyroid tissue is still important to ensure therapeutic efficacy.

Iodinated contrast media (ICM) is often used in DTC patients with locally aggressive disease or clinically apparent cervical lymph node to optimize preoperative planning and the completeness of surgery (4). Because ICM contains several 100-fold the recommended daily allowance of iodine and may cause a retention of iodine in the body for years (5, 6), there has long been a concern among nuclear medicine physicians that it could interfere with thyroid RAI uptake. Accordingly, in clinical practice, RAI administration was usually delayed for a certain period to eclipse this effect (7, 8). However, when a low-dose RAI protocol is applied, the interference of preoperative use of ICM may become significantly pronounced. It is possible that the patients' clinical outcome and management strategy will be altered in this scenario.

Response-to-therapy assessment during the first 1–2 years after initial therapy for DTC patients is effective in estimating risk of long-term recurrence and was endorsed by the 2015 American Thyroid Association (ATA) guidelines (4). The most significant impact of this system is in patients with excellent response (ER), in whom the risk of disease recurrence was very low (1–4%), and far less intensive management would be required during follow-up. Thus, it is desirable to ensure that patients have a better chance of ER after RAI therapy.

In the present study, we evaluated whether the ER rates were influenced by preoperative use of ICM in the setting of a low-dose RAI protocol. Patients with initial ATA intermediate risk of recurrence were included, in whom the risk of recurrence is significant and in whom the impact of the response to therapy is most evident in terms of follow-up.

## Materials and Methods

### Patients

The China-Japan Union Hospital is a tertiary-care University teaching center in northeastern China providing comprehensive care for thyroid cancer patients. All DTC patients in our center, except those with primary tumor measuring 1 cm or less confined to the thyroid gland, underwent total thyroidectomy (TT) with central or lateral neck dissection, depending on risk and intraoperative findings. From August 2016 on, the thyroid surgery department began to routinely select DTC patients to perform preoperative contrast CT following the 2015 ATA guidelines. However, because these guidelines also indicated that a 4–8-week interval between the use of ICM and RAI administration, which was defined as “RAI delay time” in this article, would be adequate to eclipse the impact of ICM on RAI therapy, in our department, the RAI delay time for all patients (with or without ICM) in this period were solely determined by the patient's access to medical facilities and availability of RAI for administration.

**Figure 1:**
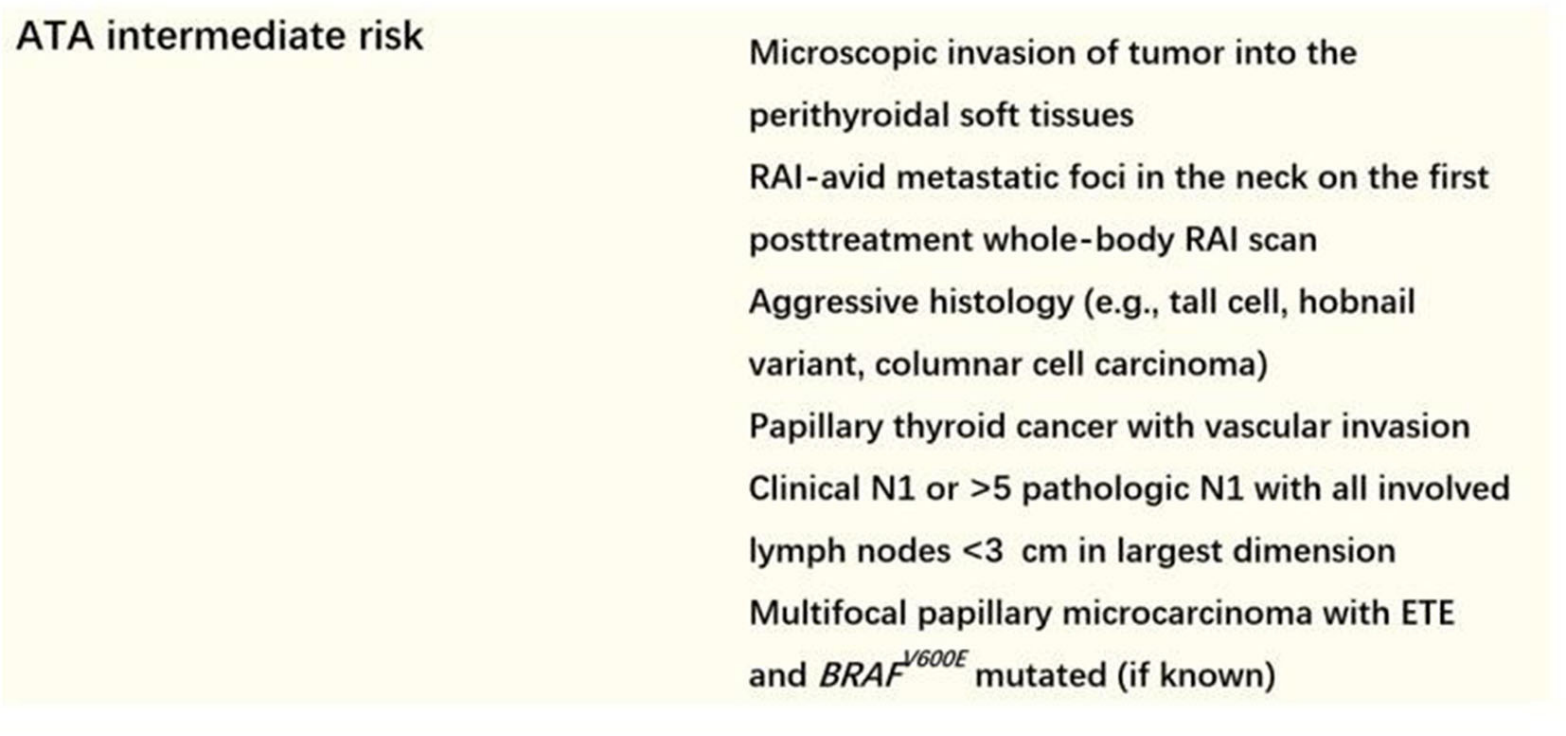
Criteria of 2015 ATA intermediate-risk patients.

We screened ATA intermediate-risk patients (detailed definition of intermediate risk in the 2015 ATA recurrence stratification system was shown in Figure 1) who had undergone TT and low-dose RAI therapy between August 2016 and August 2018. Patients who met the following criteria were excluded: (1) RAI delay time was more 6 months; (2) suspicion of distant metastases because of elevated serum ps-Tg level, radiological findings including chest computed tomography (CT), or positron emission tomography/CT, or therapeutic RAI scan, or histopathological biopsy; (3) positive or elevated serum Tg antibody (TgAb) level; and (4) patients with incomplete clinical data. Finally, a total of 683 patients were retrospectively enrolled. The study was approved by the local ethics committee.

### Preoperative ICM and Measurement of Preablation Urinary Iodine Concentration

Preoperative ICM included iodixanol, iohexol, and iopromide, which are all non-ionic and have an iodine concentration of 320, 350, and 370 mg/mL, respectively. The ICM dose administered was 100 mL for iodixanol or iohexol and 80–100 mL for iopromide per CT scan.

Preablation urinary iodine (UI) concentration was measured using a rapid kit (Zhongsheng Jinyu Diagnostic Technology Company Limited, Beijing, China) developed based on the study of Rendl et al. (9, 10). After sample collection (between 8 and 11 am), measurement was done within 2 h according to manufacturer's protocol.

### RAI Protocol

Patients were prepared by levothyroxine (LT4) withdrawal together with a strict low-iodine diet for at least 2 weeks, with the goal of attaining an appropriate thyroid-stimulating hormone (TSH) level > 30 μIU/mL. Patients who were scheduled to perform an imminent RAI therapy should have a UI concentration of <200 μg/L. The RAI dose administered was based on the 2015 ATA guidelines according to TNM stage and recurrence risk stratification. For intermediate-risk patients included in this study, a fixed low-dose RAI was administered for successful remnant ablation (Table 1 shows the protocol for RAI dosing in our department). Thyroxine therapy was resumed on the third day, and a therapeutic RAI scan was performed 3–5 days after RAI therapy.

**Table 1:** Protocol for RAI dosing.

**Risk stratification**	**Aim**	**Dose**
Low and intermediate risk^*^	Remnant ablation	1,110 MBq
High risk	Adjuvant therapy	3,700–5,550 MBq
	Therapy for persistent disease	3,700–7,400 MBq

* *For patients with low risk of recurrence, RAI was administered based on patient's preference, although the ATA guideline did not routinely recommend RAI therapy for these patients*.

### Response Assessment

Response assessment was performed 16–40 months after RAI therapy. In this study, the response was divided into ER and NER according to the serological examination (suppressed Tg, stimulated Tg, and TgAb) and imaging technique (DxWBS, cervical ultrasound, chest CT, and bone scintigraphic imaging) described in the 2015 ATA guidelines. Excellent response was defined as negative imaging and at the same time either suppressed Tg up to 0.2 ng/mL or ps-Tg up to 1 ng/mL with the absence of TgAb.

### Statistical Analysis

We utilized propensity score methods to adjust for difference in the baseline characteristics of patients in the ICM and non-ICM groups. To estimate the propensity score, logistic regression was performed with three variables: T stage, N stage, and UI (all *P* < 0.001 in univariate analyses). After propensity score estimation, the ICM and non-ICM groups were matched according to propensity score in a 1:1 ratio with a caliper of 0.05. Univariate analyses were performed to compare the clinical characteristics of the patients and ER rates between the two groups. To evaluate the impact of preoperative use of ICM on ER rates, non-adjusted, risk-adjusted, and fully adjusted logistic regression models were constructed for the both primary and the matched cohort. For risk-adjusted analyses, we adjusted for features that were significant in the univariate analyses. The subgroup analyses were performed using R × C χ^2^ test. Categorical variables were compared with either χ^2^ test or Fisher exact test as appropriate. Student *t*-test was used for normally distributed continuous variables, and the Mann-Whitney *U*-test was used for non-normally distributed continuous variables. Statistical analysis was performed using R software (version 3.4.3; http://www.R-project.org, The R Foundation). *P* < 0.05 was considered to indicate statistical significance.

## Results

### Patients

For the included patients, five hundred and thirty-two of them underwent preoperative ICM and 151 did not. Patients' baseline characteristics are shown in Table 2. As can be seen, T stage (*P* < 0.001), N stage (*P* < 0.001), UI (*P* < 0.001), and ps-Tg (*P* = 0.042) in the ICM group were significantly higher than that of the non-ICM group. After propensity score matching, 93 pairs of patients were successfully matched, and all baseline characteristics were well-balanced between the matched groups.

**Table 2:** Patients' baseline characteristics.

	**Primary cohort (*****n=*** **683)**			**Matched cohort (*****n=*** **186)**		
	**Non-ICM (*n =* 532)**	**ICM (*n =* 151)**	***P*-value**	**Non-ICM (*n =* 93)**	**ICM (*n =* 93)**	***P*-value**
Age at diagnosis [n (%)]	41.5 ± 14.8	41.5 ± 13.0	0.989	38.8 ± 13.9	42.1 ± 13.6	0.105
Gender [n (%)]			0.138			0.213
Female	362 (68.0%)	93 (61.6%)		66 (71.0%)	58 (62.4%)	
Male	170 (32.0%)	58 (38.4%)		27 (29.0%)	35 (37.6%)	
Multifocality [n (%)]			0.228			0.552
No	230 (43.2%)	57 (37.7%)		41 (44.1%)	37 (39.8%)	
Yes	302 (56.8%)	94 (62.3%)		52 (55.9%)	56 (60.2%)	
T stage [n (%)]			<0.001			0.069
T1	335 (63.0%)	55 (36.4%)		47 (50.5%)	55 (59.1%)	
T2	86 (16.2%)	2 (1.3%)		9 (9.7%)	1 (1.1%)	
T3	94 (17.7%)	80 (53.0%)		28 (30.1%)	29 (31.2%)	
T4	17 (3.2%)	14 (9.3%)		9 (9.7%)	8 (8.6%)	
N stage [n (%)]			<0.001			0.247
N0	86 (16.2%)	7 (4.6%)		5 (5.4%)	7 (7.5%)	
N1a	347 (65.2%)	31 (20.5%)		42 (45.2%)	31 (33.3%)	
N1b	99 (18.6%)	113 (74.8%)		46 (49.5%)	55 (59.1%)	
Delay time [n (%)]			0.742			0.883
1–2 months	100 (18.8%)	27 (17.9%)		14 (15.1%)	18 (19.4%)	
2–3 months	211 (39.7%)	54 (35.8%)		35 (37.6%)	31 (33.3%)	
3–4 months	97 (18.2%)	33 (21.9%)		16 (17.2%)	18 (19.4%)	
4–5 months	82 (15.4%)	22 (14.6%)		19 (20.4%)	16 (17.2%)	
5–6 months	42 (7.9%)	15 (9.9%)		9 (9.7%)	10 (10.8%)	
Histologic subtype [n (%)]			0.980			0.601
Papillary	500 (94.0%)	142 (94.0%)		86 (92.5%)	84 (90.3%)	
Follicular	32 (6.0%)	9 (6.0%)		7 (7.5%)	9 (9.7%)	
^99m^Tc-pertechnetate uptake [n (%)]			0.187			0.240
Negative	253 (47.6%)	81 (53.6%)		40 (43.0%)	48 (51.6%)	
Positive	279 (52.4%)	70 (46.4%)		53 (57.0%)	45 (48.4%)	
TSH (μIU/mL), mean	103.6 ± 34.9	99.1 ± 28.1	0.151	101.1 ± 34.3	100.5 ± 28.5	0.895
Ps-Tg (ng/ml), mean	4.3 ± 4.2	5.1 ± 4.6	0.042	4.6 ± 4.2	5.6 ± 4.7	0.122
UI (μg/L), mean	82.9 ± 30.9	96.5 ± 32.1	<0.001	95.6 ± 36.0	91.1 ± 30.4	0.356

### Impact of Preoperative Use of ICM on ER Rates

The distribution of ER rates for the ICM patients and the non-ICM patients is shown in Table 3. Excellent response rates in the ICM group were lower than those of the non-ICM group either in the primary cohort (53.6 vs. 65.0%) or in the matched cohort (45.7 vs. 54.3%).

**Table 3:** Factors associated with the excellent response.

	**Primary cohort (*****n=*** **683)**			**Matched cohort (*****n=*** **186)**		
	**NER (*n =* 256)**	**ER (*n =* 427)**	***P*-value**	**NER (*n =* 81)**	**ER (*n =* 105)**	***P*-value**
ICM [n (%)]			0.011			0.183
No	186 (72.7%)	346 (81.0%)		36 (44.4%)	57 (54.3%)	
Yes	70 (27.3%)	81 (19.0%)		45 (55.6%)	48 (45.7%)	
Age at diagnosis (years), mean	41.0 ± 15.1	41.8 ± 14.0	0.462	39.3 ± 14.4	41.3 ± 13.3	0.344
Gender [n (%)]			0.360			0.530
Female	176 (68.8%)	279 (65.3%)		52 (64.2%)	72 (68.6%)	
Male	80 (31.2%)	148 (34.7%)		29 (35.8%)	33 (31.4%)	
Multifocality [n (%)]			0.372			0.074
No	102 (39.8%)	185 (43.3%)		28 (34.6%)	50 (47.6%)	
Yes	154 (60.2%)	242 (56.7%)		53 (65.4%)	55 (52.4%)	
T stage [n (%)]			0.958			0.24
T1	146 (57.0%)	244 (57.1%)		51 (63.0%)	51 (48.6%)	
T2	32 (12.5%)	56 (13.1%)		4 (4.9%)	6 (5.7%)	
T3	65 (25.4%)	109 (25.5%)		21 (25.9%)	36 (34.3%)	
T4	13 (5.1%)	18 (4.2%)		5 (6.2%)	12 (11.4%)	
N stage [n (%)]			0.194			0.664
N0	32 (12.5%)	61 (14.3%)		5 (6.2%)	7 (6.7%)	
N1a	134 (52.3%)	244 (57.1%)		29 (35.8%)	44 (41.9%)	
N1b	90 (35.2%)	122 (28.6%)		47 (58.0%)	54 (51.4%)	
Delay time [n (%)]			0.175			0.954
1–2 months	47 (18.4%)	80 (18.7%)		16 (19.8%)	16 (15.2%)	
2–3 months	88 (34.4%)	177 (41.5%)		28 (34.6%)	38 (36.2%)	
3–4 months	48 (18.8%)	82 (19.2%)		14 (17.3%)	20 (19.0%)	
4–5 months	47 (18.4%)	57 (13.3%)		15 (18.5%)	20 (19.0%)	
5–6 months	26 (10.2%)	31 (7.3%)		8 (9.9%)	11 (10.5%)	
Histologic subtype [n (%)]			0.833			0.986
Papillary	240 (93.8%)	402 (94.1%)		74 (91.4%)	96 (91.4%)	
Follicular	16 (6.2%)	25 (5.9%)		7 (8.6%)	9 (8.6%)	
^99m^Tc-pertechnetate uptake [n (%)]			<0.001			0.002
Negative	99 (38.7%)	235 (55.0%)		28 (34.6%)	60 (57.1%)	
Positive	157 (61.3%)	192 (45.0%)		53 (65.4%)	45 (42.9%)	
TSH (μIU/mL), mean	102.8 ± 33.7	102.5 ± 33.5	0.916	101.2 ± 34.0	100.5 ± 29.5	0.878
Ps-Tg (ng/ml), mean	7.2 ± 4.9	3.6 ± 3.6	<0.001	7.0 ± 4.5	3.6 ± 3.8	<0.001
UI (μg/L), mean	86.7 ± 29.5	85.4 ± 32.9	0.172	91.9 ± 30.2	94.5 ± 35.6	0.595

In univariate analyses, for the primary cohort, significant difference was found between ER group and non-ER group for the use of ICM (*P* = 0.011), ^99m^Tc-pertechnetate uptake (*P* < 0.001) and ps-Tg (*P* < 0.001); for the matched cohort, significant difference was found between the ER group and non-ER group for the use of ICM (*P* = 0.011), ^99m^Tc-pertechnetate uptake (*P* = 0.002), and ps-Tg (*P* < 0.001) (Table 3). In multivariate analyses, the use of ICM was found to be significantly associated with decreased ER rates in crude model [odds ratio (OR) = 0.61, 95% confidence interval (CI) = 0.43–0.90, *P* = 0.011, in the primary cohort; and OR = 0.67, 95% CI = 0.38–1.21, *P* = 0.184, in the matched cohort], risk-adjusted model (OR = 0.47, 95% CI = 0.32–0.71, *P* < 0.001, in the primary cohort; and OR = 0.48, 95% CI = 0.25–0.94, *P* = 0.031, in the matched cohort), and fully adjusted model (OR = 0.48, 95% CI = 0.29–0.80, *P* = 0.005, in the primary cohort; and OR = 0.51, 95% CI = 0.25–1.04, *P* = 0.065, in the matched cohort) (Table 4).

**Table 4:** Multivariate regression for impact of preoperative ICM on ER rates.

	**Crude model**		**Risk adjusted model**		**Fully adjusted model**	
	**OR/β (95%CI)**	***P*-value**	**OR/β(95%CI)**	***P*-value**	**OR/β (95%CI)**	***P*-value**
Primary cohort						
Non-ICM	Reference		Reference		Reference	
ICM	0.61 (0.43, 0.90)	0.011	0.47 (0.32, 0.71)	<0.001	0.48 (0.29, 0.80)	0.005
Matched cohort						
Non-ICM	Reference		Reference		Reference	
ICM	0.67 (0.38, 1.21)	0.184	0.48 (0.25, 0.94)	0.031	0.51 (0.25, 1.04)	0.065

*Crude model: non-adjusted; Risk adjusted model: ^99m^Tc-pertechnetate uptake and ps-Tg were adjusted in the primary cohort and matched cohort*.

*Fully adjusted model: all factors were adjusted*.

### Subgroup Analysis on RAI Delay Time

The RAI delay time of the 683 patients were categorized into five subgroups, and the number of patients in each group can be seen in Table 2. In the primary cohort, the ER rates in ICM patients were significantly lower than those of non-ICM patients for 1–2 months (*P* = 0.0245) and >2–3 months (*P* = 0.0221) subgroups, but not for >3–4 months, >4–5 months, and >5–6 months subgroups (All *P* > 0.05) (Table 5).

**Table 5:** Subgroup analysis on RAI delay time in the primary cohort.

**RAI delay time**		**Non-ICM patients**	**ICM patients**	***P*-value**
1–2 months	ER	68 (68.00%)	12 (44.44%)	0.024
	NER	32 (32.00%)	15 (55.56%)	
2–3 months	ER	148 (70.14%)	29 (53.70%)	0.022
	NER	63 (29.86%)	25 (46.30%)	
3–4 months	ER	61 (62.89%)	21 (63.64%)	0.939
	NER	36 (37.11%)	12 (36.36%)	
4–5 months	ER	45 (54.88%)	12 (54.55%)	0.978
	NER	37 (45.12%)	10 (45.45%)	
5–6 months	ER	24 (57.14%)	7 (46.67%)	0.484
	NER	18 (42.86%)	8 (53.33%)	

### Relationship Between UI Concentration and Clinical Outcomes

In univariate and multivariate analyses, UI concentration was found to be not associated with ER rates for either the primary or the matched cohort (All *P* > 0.05). Table 6 shows UI concentration according to clinical outcomes for the five RAI delay time subgroups in the primary cohort. Still, no association between UI concentration and ER rates was found within each of the five subgroups (All *P* > 0.05).

**Table 6:** UI concentration according to clinical outcomes for the five RAI delay time subgroups in the primary cohort.

**RAI delay time**	**UI in ICM patients**			**UI in non-ICM patients**		
	**ER**	**NER**	***P*-value**	**ER**	**NER**	***P*-value**
1–2 months	110.8 ± 39.6	100.7 ± 23.7	0.416	80.3 ± 28.4	80.9 ± 25.4	0.913
>2–3 months	99.0 ± 33.8	98.4 ± 31.2	0.950	84.3 ± 31.0	87.6 ± 27.6	0.457
>3–4 months	98.6 ± 33.4	85.8 ± 31.2	0.289	85.8 ± 35.5	82.1 ± 28.5	0.605
>4–5 months	88.3 ± 32.7	90.0 ± 26.7	0.899	75.3 ± 32.2	81.9 ± 32.0	0.360
>5–6 months	97.1 ± 33.5	82.5 ± 35.4	0.427	79.2 ± 36.3	84.1 ± 29.1	0.509

## Discussion

The 2015 ATA guidelines assumed that the use of ICM should not be a major concern for RAI therapy as long as 4–8 weeks' delay time was fulfilled (4). However, this arbitrary cutoff was only inferred based on the clearance time of UI values (11–13). It is unclear whether these values accurately reflect free iodide accumulation by thyroid tissue. Moreover, in a recent study, Vassaux et al. suggested that independent of free iodide, the ICM itself could directly reduce thyroid iodide uptake by decreasing NIS expression in thyroid cells. Besides, they found that ICM induces thyroid stunning to a greater and longer-lasting degree than free iodide found in ICM could explain (14). Thus, the influence of the use of ICM might have been stronger and more enduring than we previously thought. Nevertheless, although the interference of ICM on thyroid RAI uptake is well-documented in the literature (15), as far as we know, there have been no previous studies to directly and systemically evaluate the impact of preoperative use of ICM on patients' clinical outcome after RAI therapy.

In the present study, by analyzing a large cohort of intermediate-risk patients in the setting of a low-dose RAI protocol, which our department had been implementing since August 2015 (16), we evaluated whether preoperative use of ICM significantly impacted the ER rates. We chose ER rates as a clinical endpoint in this study, because in intermediate-risk patients, ER decreases the estimated risk of recurrence from 20 to 30% to <5%, leading to less intensive follow-up and no need for TSH suppression (17). Propensity score matching was performed to balance the confounding factors in the primary cohort. In multivariate analysis, we found that the use of ICM was significantly associated with decreased ER rates in non-adjusted, risk-adjusted, and fully adjusted models for either the primary or the matched cohort. This implies that the use of ICM has a significant negative impact in terms of patients' clinical outcome, although it might help to improve evaluation of tissue planes and detection of local invasion before surgery. Thus, the negative impact of ICM on RAI therapy cannot be ignored in this scenario, and decision making of the use of ICM should at least take into account whether the patients are likely to receive low-dose RAI therapy afterward. To go a step further, withholding ICM might be an option in patients with lower tumor burden. As a matter of fact, toward the necessity of preoperative use of ICM, many scholars have already argued that CT without ICM can provide sufficient information regarding tumor and vascular and aerodigestive structures for effective surgical planning (18).

**Figure 2:**
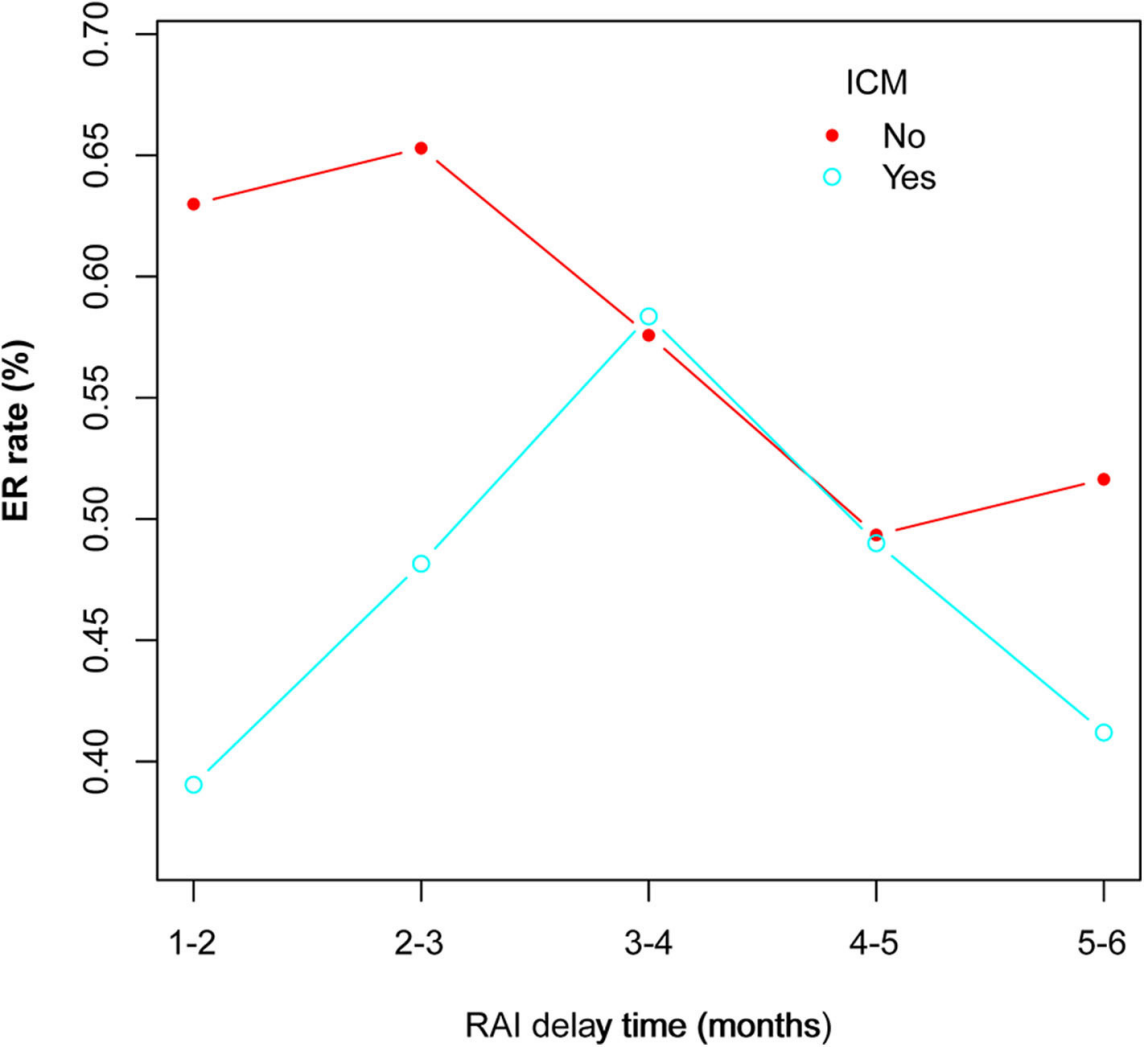
Changes of ER rates as RAI delay time extends in both ICM and non-ICM patients.

In a subgroup analysis on RAI delay time, we showed that in the primary cohort ER rates in ICM patients were significantly lower than that of non-ICM patients for 1–2 months (*P* = 0.0245) and >2–3 months (*P* = 0.0221) subgroups, but not for >3–4 months, >4–5 months, and >5–6 months subgroups (All *P* > 0.05). This indicated that the would-be negative impact of ICM on RAI therapy might persist until 3 months after surgery. In other words, if the patients had received contrast CT, the RAI delay time should be at least more than 3 months for the ICM not to significantly influence ER rates. Moreover, the RAI delay time itself is believed to impact RAI therapy, and longer delay time usually correlates with worse clinical outcome (19, 20). In our ICM group, the ER rates showed a first increased and then decreased trend among the five delay time subgroups, with the >3–4 months subgroup exhibiting the highest (Figure 2). It could be speculated that this time period might be able to better balance the decreasing negative impact of ICM and the increasing negative impact of delay time itself on RAI therapy. Thus, an RAI delay time of >3–4 months seems to be more appropriate for patients to achieve better ER rates in this scenario.

Urinary iodine is an easily obtainable indicator for iodine status and a sensitive marker of iodine intake and changes in iodine status. Previously, several researchers concluded that 1-month delay is sufficient for ICM patients to perform RAI therapy because UI concentration could return to baseline values (before the use of ICM) during this period (11, 12). However, in our study, although the UI concentrations of all included patients were within normal range (<200 μg/L) before RAI therapy, it was found to be not associated with the clinical outcome for either the primary or the matched cohort or within each of the five RAI delay time subgroups in the primary cohort. These results indicated that UI concentration within normal range cannot guarantee the absence of interference from ICM in terms of clinical outcome. This was in accordance with the theory proposed by Vassaux et al. (14) in *in vitro* studies as mentioned previously. Therefore, our study demonstrated in a clinical practice setting that it might be not suitable for using a “normal UI concentration” to decide the initiation of RAI therapy if the patients had received preoperative ICM.

There are some limitations to this study. First, it was a retrospective study performed in a single institution. Although propensity score matching was used to minimize the effect of observed confounders, it cannot address unobserved confounders. For example, preoperative contrast CT was performed at the surgeons' discretion after seeing the result of the neck ultrasound. This could have already led to selection bias. In this regard, a multi-institutional prospective randomized trial with a larger number of patients would be more appropriate. Second, the follow-up duration in our study is relatively short, and continued observations are still needed to evaluate long-term clinical outcomes. Lastly, we used a rapid UI test method that is not completely quantitative, and rather than a period of 24 h, only single-spot urinary was collected. These might undermine the results derived from UI concentration in our study.

## Conclusions

Preoperative use of ICM is associated with decreased ER rates in intermediate-risk DTC patients who subsequently receive TT and low-dose RAI therapy. For such patients, if ICM has already been received, an RAI delay time of >3–4 months would seem to be more appropriate to achieve better ER rates.

## Data Availability Statement

The raw data supporting the conclusions of this article will be made available by the authors, without undue reservation.

## Ethics Statement

The studies involving human participants were reviewed and approved by ethics committee of China-Japan Union Hospital. Written informed consent for participation was not required for this study in accordance with the national legislation and the institutional requirements.

## Author Contributions

JB, MQ, and WL conceived and designed the study. WL, WR, WQ, and TF performed the retrospective study. WR and WQ interpreted the data. WL and JB wrote the paper. JB and MQ supervised the study, reviewed, and edited the manuscript. All authors approved the final manuscript.

## Conflict of Interest

The authors declare that the research was conducted in the absence of any commercial or financial relationships that could be construed as a potential conflict of interest.
